# PM_2.5_ exposure induces functional alterations in pregnant rats heart and in human stem cell derived cardiac spheroids

**DOI:** 10.1007/s00204-026-04337-8

**Published:** 2026-03-06

**Authors:** Flavia Bonalumi, Margherita Burattini, Rosario Statello, Mirko Hu, Minh Long Hoang, Nicola Delmonte, Alessia Caputo, Barbara Montanini, Francesco Paolo Lo Muzio, Silvana Pinelli, Paola Mozzoni, Jessica Modica, Andrea Cattaneo, Francesca Rossi, Enrico Bergamaschi, Valentina Bollati, Stefano Rossi, Michele Miragoli

**Affiliations:** 1 Department of Medicine and Surgery, University of Parma, Parma, Italy; 2 Department of Surgery, Dentistry, Pediatrics and Gynaecology, University of Verona, Verona, Italy; 3 Department of Engineering and Architecture, University of Parma, Parma, Italy; 4 Department of Chemistry, Life Sciences and Environmental Sustainability, University of Parma, Parma, Italy; 5 Interdepartmental Research Centre Biopharmanet-Tec, University of Parma, Parma, Italy; 6 Charité Universitätsmedizin Berlin, Berlin, Germany; 7 CERT, Center of Excellence for Toxicological Research, University of Parma, Parma, Italy; 8 IRCCS- Humanitas Research Hospital, Rozzano, Milan Italy; 9 Department of Science and High Technology, Università of Insubria, Como, Italy; 10 IMEM-CNR Institute, Parma, Italy; 11 Department of Sciences of Public Health and Pediatrics, University of Turin, Turin, Italy; 12 Department of Clinical Sciences and Community Health, University of Milan, Milan, Italy; 13 Institute of Genetic and Biomedical Research, National Research Council of Italy, Milan, Italy

**Keywords:** Air pollution, Po Valley, Hypertension, *In-vivo*/*In-vitro* cardiac models, Pregnancy

## Abstract

Air pollution is a major cardiovascular risk factor, with particulate matter (PM) posing significant threats. The Po Valley remains among Europe’s most polluted areas. While PM₂.₅ is linked to cardiac dysfunction, its effects during pregnancy—especially under hypertensive conditions—are poorly defined. We investigated how prolonged PM exposure from Milan’s urban area affects cardiac electromechanical function in pregnant normotensive and hypertensive rats, and in a human embryonic stem cell-derived 3D cardiac spheroid. Pregnant normotensive and hypertensive (SHR) rats were exposed to saline solutions w/wo PM (2 mg/kg) three times weekly for 19 days. Cardiac spheroids were cultured in DMEM w/wo PM (10–50 µg/mL) for 8 days. We assessed cardiac function via *in-vivo* epicardial mapping and *in-vitro* optokinematic analysis. Inflammatory, toxicological, and molecular profiles were evaluated. Machine learning classified electrogram profiles in PM-exposed versus unexposed rats. In SHR rats, PM exposure increased rheobase (+ 39%), current threshold (+ 32%), and chronaxie (+ 71%), with a 21% rise in effective refractory period. Conduction velocity and anisotropy were also altered. Machine learning achieved > 86% and > 77% classification accuracy in normotensive and SHR, respectively. In human cardiac spheroids derived from embryonic stem cells, short-term PM exposure impaired relaxation (− 36% at 10 µg/mL; − 21.5% at 20 µg/mL) and contraction amplitude (− 42% and − 62%, respectively). Long-term evaluation disrupted calcium transients (− 20% and − 15%) and altered IL-6. Gene expression revealed dysregulation of calcium-handling genes. These findings emphasize the heightened risks of PM exposure during gestation, particularly in preeclampsia, and support the need for public health strategies to protect maternal and fetal cardiovascular health.

**Supplementary Information:**

The online version contains supplementary material available at 10.1007/s00204-026-04337-8.

## Introduction

The number of pregnancies affected by hypertensive disorders (HDP) and related complications has been rising steadily over the last three decades (Abalos et al. 2013). While this concerning trend has been addressed in literature through multiple observational studies and meta-analyses (Ferrari et al. 2020; Pedersen et al. 2013; Simoncic et al. 2020) attempting to identify causative factors, the role of environmental toxicants is not fully understood yet.

During pregnancy, the cardiovascular system undergoes profound adaptations that may be disrupted by environmental insults (Abalos et al. 2013; Márquez-Lázaro et al. 2024). Among these, ambient air pollution—especially particulate matter (PM) with aerodynamic diameter ≤ 2.5 µm—has emerged as a significant risk factor for HDPs and adverse maternal outcomes (Podjarny et al. 1997).

Chronic PM exposure is known to induce cardiovascular dysfunctions through both structural remodeling and electrophysiological disturbances (Culic 2014; Lacasa et al. 2008; Lo Muzio et al. 2020; Nemmar et al. 2003). Moreover, exposure to ambient PM, which can accumulate on the fetal side of placenta (Bove et al. 2019) and result in placental pathological changes (Liu et al. 2016), has been linked to preterm birth, low birth weight, and growth restriction (Cattaneo et al. 2021; Mozzoni et al. 2022; Rossi et al. 2023). In highly polluted regions, PM exposure during the periconceptional period has even been related to congenital heart defects (Yuan et al. 2023).

However, PM cardiotoxicity is not comprehensively described during gestation, especially when HDPs occur simultaneously. Besides, the impact of PM on cardiomyocytes should also be thoroughly investigated in immature/developing cardiac cells. Importantly, the physicochemical properties of PM_2.5_ vary significantly across different geographical regions, suggesting the need for new insightful studies on this topic.

In this study, PM_2.5_ from Milan’s urban area (PM-Mi) in the Po Valley, one of Europe’s most polluted regions, was characterized. Subsequently, it was subacutely administered to both pregnant normotensive (NR) and pregnant spontaneously hypertensive rats (SHR), that were selected to model maternal cardiovascular adaptations, with the SHR strain recapitulating HDP. Human embryonic stem cell derived cardiac spheroids were also exposed to PM-Mi. Electrophysiological, mechanical and molecular evaluations were performed to assess the effects of PM-Mi in both *in-vivo* and *in-vitro* experiments. Moreover, we evaluated the accuracy of a machine learning algorithm in classifying pregnant rats as either exposed or non-exposed to PM-Mi using a network science (NS) based analysis of electrogram (EGs) tracings (Hernandez-Lemus et al. 2024).

## Material and methods

### PM-Mi physical characterization and preparation

PM-Mi was collected and chemically characterized as reported in (Rovelli et al. 2020). Briefly, PM_2.5_ was collected by a high-volume air sampler on polytetrafluoroethylene-coated glass fiber filters, extracted in ultrapure water by sonication in a cold water-bath and evaporated to dryness using a rotary evaporator. Due attention was paid to the sterility of the extraction process. PM-Mi masses were determined by gravimetric analysis. PM-Mi morphology has been characterized using Scanning Electron Microscopy (SEM) in a Zeiss Auriga Compact field emission microscope (Zeiss, IT). Physical PM-Mi characteristics as ζ-potential (electrical potential that exists at the shear plane of a particle), ζ-average (intensity-weighted mean hydrodynamic size), and polydispersity index (PDI, measure of the heterogeneity of particle or molecule sizes within a sample) were determined using dynamic light scattering (DLS, ZetaSizer Ultra, Malvern Panalytical, Malvern, UK) in 50 µg/mL resuspended in different solutions. Briefly, PM-Mi was resuspended in distilled water (dH_2_O), in saline physiological solution (NaCl 0.9%) and Dulbecco’s modified medium (DMEM, Gibco™, ThermoFisher Scientific, USA) supplemented with 10% fetal bovine serum (FBS, Biowest, France) (DMEM10). For DMEM10, any change in physical characteristics depending on the PM-Mi resuspension time was inspected after 24 h, 7 days and one month. For the *in-vitro* and *in-vivo* experiments, the PM-Mi was weighed and sterilized with ethanol (70% EtOH) under the laminar flow hood. Once EtOH evaporated completely, the PM-Mi was resuspended in either DMEM10 (1 mg/ml) or physiological solution (10 mg/mL) for the *in-vitro* and *in-vivo* respectively.

### Ethics statement and animals

Experiments were conducted on 10-weeks old Wistar female rats (NR, n = 10) and Spontaneous Hypertensive female Rats (SHR, n = 19) under standard conditions. This study was carried out following the recommendations in the Guide for the Care and Use of Laboratory Animals of the National Institute of Health (Bethesda, MD, USA, revised 1996), and the European Guideline on animal experiments (Directive 2010/63/EU). The protocol was approved by the Veterinary Animal Care and Use Committee of the University of Parma (Permit: 281/2017-PR and 989/2017-PR).

### In-vivo PM-Mi exposure

At the eleventh week of age, the staging of the oestrous cycle was evaluated with vaginal lavage, for more details, see the Supplementary materials. When the Oestrus phase was identified as detecting the highest cellular presence represented by leucocytes, the female was immediately mated for 1 day. After 5 days, systolic blood pressure (SBP) and diastolic blood pressure (DBP) were acquired by means of Blood Pressure System (Panlab) that provides an easy and reliable technique (tail cuff) to measure systemic blood pressure and cardiovascular parameters in rodents without any invasive catheterization, for more details, see the Supplementary materials. Afterwards, the subacute respiratory exposure was obtained via intratracheal instillation (Rossi et al. 2021). Briefly, under isoflurane anesthesia (2% in 100% oxygen) an angiocatheter was inserted into the trachea to deliver the saline solution (0.9% NaCl), with or without PM-Mi (2 mg/kg of body weight), directly into the lower respiratory tract (∼50 μl/250 g body weight). NR and SHR animals were randomly assigned to one of the following groups: NRs instilled with saline solution (NR_Physio_, n = 4); NRs instilled with saline solution + PM-Mi (NR_PM_, n = 6); SHRs instilled with saline solution (SHR_Physio,_ n = 10); SHRs instilled with saline solution + PM-Mi (SHR_PM_, n = 9). The sample used for intratracheal instillation was mixed and sonicated (Branson Ultrasonics, Danbury, CT, USA) for 10 min at 37 °C to minimize particle aggregation. Body weight (BW), blood pressure and intratracheal instillation were performed three times a week from the 5th to the 19th day of pregnancy. For more details see Supplementary materials section.

### Electromechanical investigation

On the 19th day of pregnancy, the measurement of physiological parameters was carried out after 4 h from the last PM-Mi instillation. In detail, the animals were anesthetized by intraperitoneal injection of 0.15 mg/kg BW of Domitor (Orion Corporation, Espoo, Finland) and 40 mg/kg BW of Lobotor (Merial, Tolouse, France). Additional amounts of anesthetic were administered if needed. Under artificial respiration (Rodent Ventilator 7025, Ugo Basile, Comerio, Italy), the heart was exposed through a longitudinally sternotomy and suspended in a pericardial cradle. An epicardial electrode array fabricated on a surgical gauze patch (11 × 11 grid size, 0.5 mm resolution square mesh) was used to record ventricular unipolar electrograms (EGs) during sinus rhythm and ventricular pacing. Moreover, cardiac excitability (Threshold, Rheobase and Chronaxie), refractoriness (Effective Refractory Period, ERP), conduction velocity of the electrical impulse and induced arrhythmogenesis were assessed during ventricular pacing as described in our previous work (Rossi et al. 2021, 2019). Furthermore, to evaluate cardiac kinematics, 2-s long videos with the temporal resolution of 500 fps were acquired 20 cm above the heart (1280 × 1024 pixels, Baumer HXC13, Baumer Italia S.r.l., Milano, Italy connected with full CameraLink® interface to a frame grabber acquisition board PCIe 1433 NI, Assago, Italy). The videos were elaborated by Video Spot Tracker and a custom MATLAB® algorithm (Fassina et al. 2017). In detail, the coordinates traced along the videos were analysed drawing trajectories and returning different kinematic parameters: Contractility; Force; Energy; Perimeter, corrected for the number of beats (Lo Muzio et al. 2025).

### Anatomical measurements and blood collection

After electrical and mechanical measurements, venous blood was collected in EDTA-coated tubes (Sarsted AG, Numbrecht, Germany) and plasma was separated by centrifugation (2600 g, 4 °C, 10 min) and stored at -80 °C. The heart and the tibia were rapidly excised. Heart weight (HW) and tibial length (TL) were measured, and HW-to-BW and HW-to-TL ratios were calculated as index of cardiac hypertrophy.

### Network science and machine learning classification

NS was applied to EGs acquired during spontaneous sinus rhythm to extract features used for classifying animals as either exposed or non-exposed to PM-Mi. Briefly, each signal was transformed into the corresponding visibility graph (Lacasa et al. 2008). Then, the resulting graph, which included nodes (i.e., the time-points) and edges (i.e., links/connections), was partitioned into communities and the nodes were assigned to a role according to the position of the node into the community (Guimera and Nunes Amaral 2005). The frequencies of each role were used as features. The network features after being transformed into percentages were fed to the machine learning (ML) classifier (Kramer et al. 2013). The classification performances were evaluated with F1-score, accuracy, and the Area Under the Curve (AUC) of the Receiver Operating Characteristic (ROC) curve. For more details, see the Supplementary materials section.

### Cardiomyocytes differentiation and long-term spheroid culturing

Rockefeller University Embryonic Stem (RUES) cell line was kindly donated from Dr. Elisa di Pasquale (IRCCS Humanitas Clinical and Research Center). When cells reached approximately 80% confluency, induction into cardiomyocytes (RUES-CMs) was started through a commercial differentiation kit (Gibco, ThermoFisher Scientific, USA). After 21 days from the start of differentiation process, the cells were tagged for cardiomyocytes-immunomagnetic purification (Miltenyi Biotec, Germany), according to manufacturer indications. Once purified, RUES-CMs were resuspended in fresh DMEM10. The collected RUES-CMs were then centrifuged and counted. The purified RUES-CMs were seeded in 96-well round-bottom ultra-low attachment plates (Corning, USA) at 50,000 cells/well concentration. After two weeks, RUES-CMs reorganised into compact, beating structures characterized by a well-defined spheroidal shape. Half-volume medium refreshment was done every 4 days. For details about cell culture, see Supplementary materials section.

### *In-vitro* PM-Mi exposure

After 21 days from seeding, cardiac spheroids were exposed to PM-Mi. Fresh dilutions of 10 μg/mL, 20 μg/mL and 50 µg/mL in DMEM10 with 1% Penicillin/Streptomycin were prepared. Spheroids were randomly divided into four groups: 10 µg/mL (n = 27), 20 µg/mL (n = 23), and 50 µg/mL (n = 23) of PM-Mi and a non-treated group (CTRL, n = 16). From each well, the old medium was discarded and replenished with 150 µL of PM-containing medium. In detail, wells containing 200 µL of medium were partially refreshed with 150 µL of PM-containing or fresh medium, leaving 50 µL, and an additional 100 µL was added after 4 days to compensate for evaporation, maintaining a constant volume throughout the exposure. The automatic acquisition was performed after 2 days (Short-term Evaluation, STE, 48 h) and after 8 days (Long-Term Evaluation, LTE, 192 h).

### Custom screening platform and contractility analysis

An inverted microscope (Nikon Eclipse Ts2-FL) connected to a high-speed camera (Basler, acA1920-155 um) was inserted into a cell culture incubator (Heraeus Instruments, BB-16 D-63450 Hanau) to guarantee 37 °C and 5% CO_2_ throughout the experiment (Longitudinal OptoKinematic Incubation, LOKI). The LOKI system was equipped with a motorized XY scanning stage (STANDA, 8MTF-102LS-MEn1). The videos acquired were analyzed with the built-in Image-J macro, MUSCLEMOTION (Sala et al. 2018). The contraction profile was further processed in MATLAB® and the following parameters were quantified: time of contraction (τ_rise_), time of relaxation (τ_fall_), beat duration, and maximum contraction C_max_ (Burattini et al. 2024).

### Calcium transient analysis

Spheroids were incubated for 20 min with 5 µM of Fluo-4AM dye (Life Technologies, catalogue # F14204) at the last timepoint (192 h). Calcium transient was captured in 4-s long video (Kinetix22, Teledyne photometrics^©^, USA) with an acquisition frame rate of 100 fps, 10 × magnification, binning 4 × 4. Changes in fluorescence intensity normalized for the baseline fluorescence intensity (ΔF/F_0_) have been analysed using a dedicated MATLAB® script (Fassina et al. 2022). The following parameters were extracted and compared: calcium release (τ_rise_), calcium reuptake (τ_fall_), calcium transient duration, τ_rise_ slew rate and τ_fall_ slew rate.

### Inflammatory protein quantification

ELISA assay was performed to test inflammatory response in both spheroid supernatant and rat plasma. On cell supernatant, Intercellular Adhesion Molecule-1 (ICAM-1), C-Reactive Protein (CRP) and Interleukin-6 (IL-6) quantification was performed through ELISA assay kit from eBIOSCIENCE (USA), R&D (France) and Origene (USA), respectively at both 48 h and 192 h timepoints. Similarly, ICAM-1(Rat ICAM-1/CD54, R&D system, MN, USA), CRP (Rat C-Reactive Protein/CRP, R&D system, MN, USA) and IL-6 (Rat IL-6, R&D system, MN, USA) were quantified on rat plasma collected before sacrifice. Once thawed, the samples were processed according to the manufacturer’s protocols with appropriate sample dilutions. The absorbance at 450 nm was read (Thermo Scientific™, Varioskan™ LUX multimode microplate reader, USA) and the optical density values (O.D.) were used to calculate the standard curves concentration fitted on 4-parametric calibration curves (SkanIt Software 7.0.2 RE).

### Gene expression analysis

RNA from rat heart and spheroids was extracted and used to synthesize complementary DNA (cDNA), starting from 70 ng of RNA, following the manufacturers’ instructions (see Supplementary Material). The *GAPDH* gene was used as the housekeeping gene to calculate ∆Ct, and the gene expression of each PM-treated sample was normalized to the control (ΔΔCt). The differential expression of the following genes in rat heart was evaluated: calcium voltage-gated channel subunit alpha 1C (*Cacna1C*, LTCC member), ryanodine receptor (*RyR2*), ATPase sarcoplasmic/endoplasmic reticulum Ca2 + transporting 2 (*Atp2A2*) and calcium/calmodulin-dependent protein kinase II beta (*CamIIβ*). For details please see Supplementary materials section.

### Statistical analysis

The Shapiro–Wilk test was used to check the data normality. Data were represented as mean ± standard deviation, in case of normal distribution, or median ± interquartile range (IQR), for non-normal distribution. Statistical analysis was performed with GraphPad Prism® (version 9.0) and the tests applied are indicated accordingly under each figure. For the *in-vitro* and *in-vivo* dataset, one-way ANOVA, two-way ANOVA, or Kruskal–Wallis test in case of non-normal distribution were performed. Bonferroni’s and Dunn’s post-hoc test were used to perform multiple pairwise comparisons between groups. Moreover, Fisher’s Exact Test was used to evaluate the frequencies of animals exhibiting ventricular arrhythmogenesis during the stimulation protocol for ERP evaluation. Lastly, differential genetic expression was analyzed in Excel and t-test was performed on the ΔΔCt. Statistical significance is reported as * for *p* < 0.05, ** for *p* < 0.01 and *** for *p* < 0.001.

## Results

### PM-Mi characterization

**Fig. 1:**
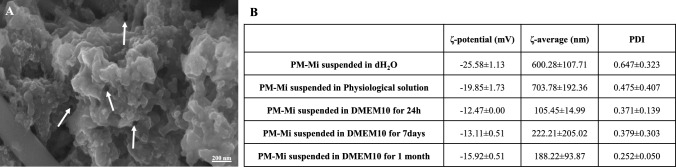
PM-Mi physical characterization **A** SEM image of PM-Mi. White arrows indicate PM-Mi nanometric porosity. Scale bar 200 nm. **B** Physical properties of PM-Mi in different biological samples. Data were reported as mean ± SD. PM-Mi: Milan suburban area particular matter; DMEM10: Dulbecco’s Modified Eagle Medium supplemented with 10% of fetal bovine serum; PDI: Polydispersity index (a.u.)

SEM revealed a highly irregular surface of PM-Mi agglomerates, which differed in size and displayed nanometric surface porosity (Fig. 1A). The physicochemical properties of PM-Mi are summarized in Fig. 1B and in Table S1. The ζ-potential remained negative in all solutions (Fig. 1B). Notably, PM-Mi suspended in DMEM10 showed a less negative ζ-potential compared to other solutions. A skew-normal size distribution of the PM-Mi was observed, even if particle size (ζ -average) peaked in physiological buffer versus DMEM10 solutions. The PDI indicated moderate aggregation tendencies across all solutions, consistent with high dispersity. The chemical composition denotes the prevalence of Fe (0.38%), Zn (0.24%), Cu (0.028%) with traces of other elements (Table S1). Forty-nine percent of the total mass, including carbonaceous and crustal particles was undetermined.

### Anatomical and physiological analysis in pregnant rats

**Fig. 2:**
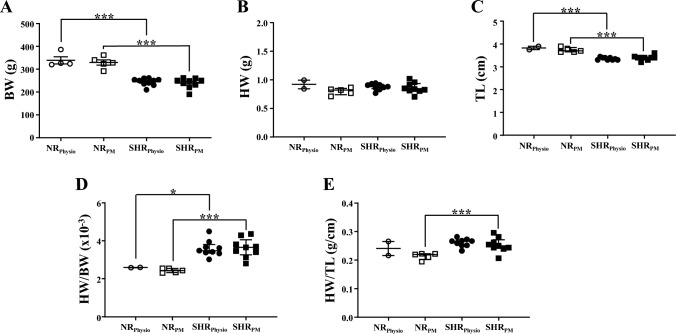
Anatomical parameters in Physio (circles) and PM-Mi (squares) treated NR (empty symbols) and SHR (filled symbols). **A** Body weight (BW), **B** Heart weight (HW), **C** Tibial length (TL), **D** heart weight on body weight ratio (HW/BW), **E** heart weight on tibial length ratio (HW/TL). NR: normotensive rats. SHR: spontaneously hypertensive rats. Data were presented as median ± interquartile range and were analyzed by by Kruskal–Wallis test. * *p* < 0.05 and *** *p* < 0.001

Compared to NR, SHR showed a significant reduction of BW and TL (Fig. 2A and C), whereas no changes were detectable for HW (Fig. 2B). The SHR group also had a significantly higher HW/BW and HW/TL, indicative of cardiac hypertrophy (Fig. 2D and E). The blood pressure measurement, evaluated at the 19th day of pregnancy, demonstrated that not only SHR animals have higher SBP and DBP than NR as expected, but PM-Mi increased significantly DBP values (Fig. S1A and S1B). No differences in blood pressure were observed during pregnancy after subacute PM-Mi administration (Fig. S1C and S1D).

**Fig. 3:**
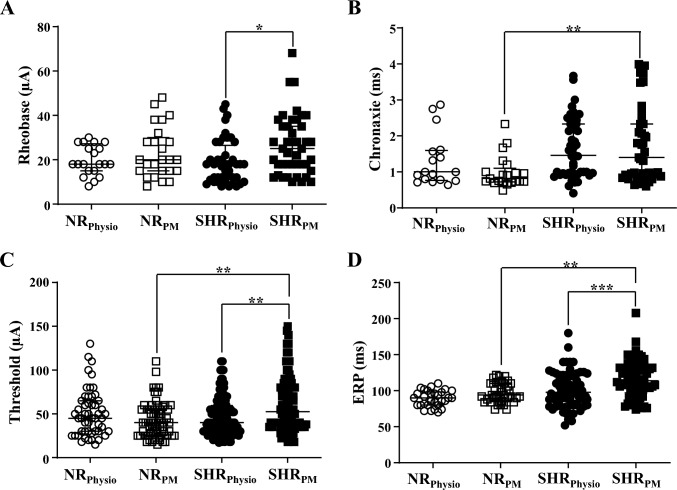
Excitability and refractoriness parameters in Physio (circles) and PM-Mi (squares) treated NR (empty symbols) and SHR (filled symbols). **A** Rheobase, **B** Chronaxie, **C** Threshold **D** effective refractory period (ERP). NR: normotensive rats. SHR: spontaneously hypertensive rats. Data were presented as median ± interquartile range and were analyzed by Kruskal–Wallis test. * *p* < 0.05, ** *p* < 0.01 and *** *p* < 0.001

The impact of PM-Mi on excitability and refractoriness parameters are presented in Fig. 3. The Rheobase increased by 39% in SHR_PM_ compared to SHR_Physio_ (SHR_Physio_: 18.0 µA IQR 34.3 µA; SHR_PM_: 25.0 µA IQR 45.0 µA) (Fig. 3A). The Chronaxie was 71% higher in SHR_PM_ compared to NR_PM_ (NR_PM_: 0.82 ms IQR 1.77 ms; SHR_PM_: 1.40 ms IQR 3.29 ms) (Fig. 3B). A 32% increment of the Threshold was detected in SHR_PM_ compared to both NR_PM_ and SHR_Physio_ (NR_PM_: 40.0 µA IQR 62.0 µA; SHR_Physio_: 40.0 µA IQR 71.8 µA; SHR_PM_: 52.5 µA IQR 100.5 µA) (Fig. 3C). Similarly, ERP increasing by 16% and 21% in SHR_PM_ compared to SHR_Physio_ and NR_PM_, respectively (NR_PM_: 94.0 ms IQR 45.7 ms; SHR_Physio_: 98.0 ms IQR 74.0 ms; SHR_PM_: 114.0 ms IQR 74.8 ms) (Fig. 3D).

**Fig. 4:**
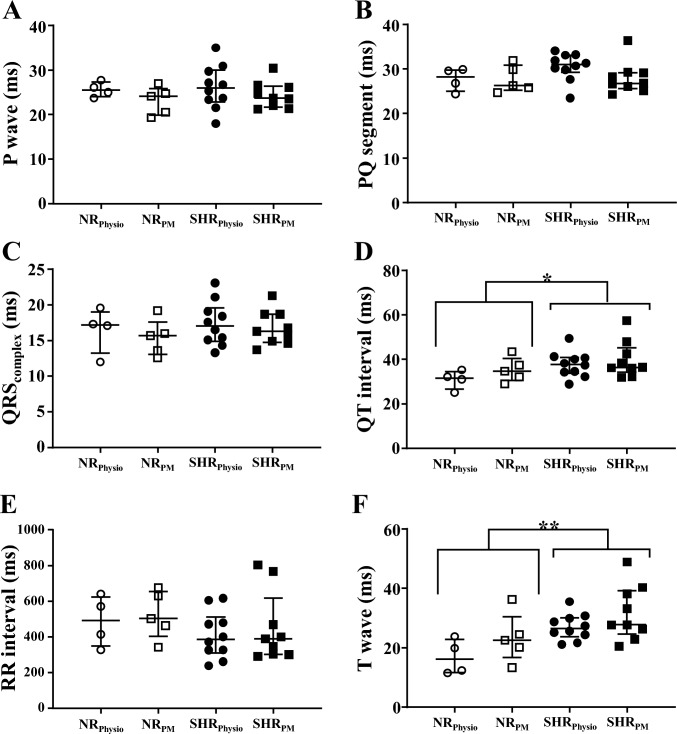
Electrogram wave, segment and interval evaluation in Physio (circles) and PM-Mi (squares) treated NR (empty symbols) and SHR (filled symbols). **A** P wave duration, **B** PQ segment duration, **C** QRS complex duration, **D** QT interval duration, **E** RR interval duration, **F** T wave duration. NR: normotensive rats. SHR: spontaneous hypertensive rats. Data were represented as median ± interquartile range and were analyzed by by Kruskal–Wallis test. * *p* < 0.05 and ** *p* < 0.01

The electrogram waves, segment and interval evaluation are displayed in Fig. 4.

**Fig. 5:**
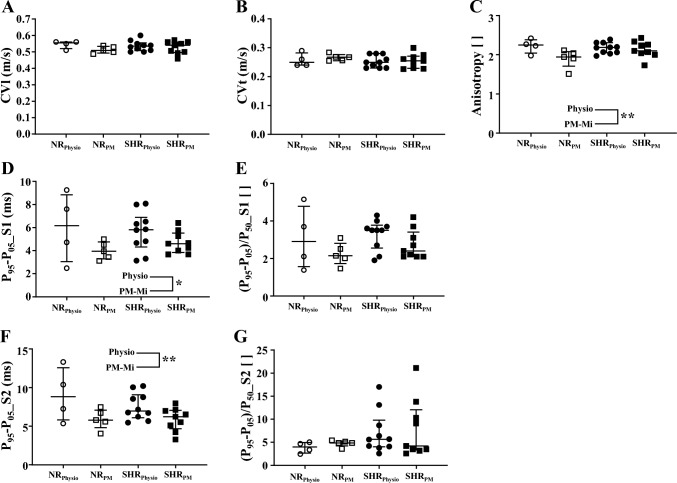
Ventricular conduction parameters in in Physio (circles) and PM-Mi (squares) treated NR (empty symbols) and SHR (filled symbols). **A** conduction velocity evaluated along epicardial longitudinal fiber direction (CV_l_), **B** conduction velocity evaluated across epicardial transverse fiber direction (CV_t_), **C** Anisotropy ratio (CV_l_/CV_t_), **D** total range in maximal differences in activation time evaluated during diastolic stimulation (P_95_-P_05__S1), **E** total range in maximal differences in activation time evaluated during premature stimulation ((P_95_-P_05_)/P_50__S1), **F** variation coefficient evaluated during diastolic stimulation P_95_-P_05__S2), **G** variation coefficient evaluated during premature stimulation (P_95_-P_05_)/P_50__S2). NR: normotensive rats. SHR: spontaneously hypertensive rats. Data were represented as median ± interquartile range and were analyzed by by Kruskal–Wallis test. *p < 0.05 and **p < 0.01

In detail, only QT interval, and T wave were prolonged in SHR group compared to NR (Fig. 4D and F). We then seek to investigate if epicardial conduction velocity was affected by the combination of PM and hypertension. Data indicated that although no differences were observed after PM-Mi treatment (Fig. 5A and B), anisotropy ratio was significantly reduced after PM-Mi administration (Fig. 5C). The phase map analysis for investigating the heterogeneity in conduction, revealed that PM-Mi exposure led to a significant reduction in P_95_-P_05__S1 and in P_95_-P_05__S2 (Fig. 5D and F). No significant changes were observed in (P_95_-P_05_)/P_50__S1 and (P_95_-P_05_)/P_50__S2 (Fig. 5E and G).

The Fisher’s exact test on the frequencies of animals exhibiting ventricular arrhythmogenesis (NR_Physio_ = 1/4; NR_PM_ = 7/10; HR_Physio_ = 2/5; SHR_PM_ = 5/9) revealed a significant association between treatment and the occurrence of induced ventricular arrhythmias, *p* < 0.05. The odds ratio indicated that PM-Mi-treated animals were 2.8 times more likely to display ventricular arrhythmias than non-treated rats in response to the epicardial electrical stimulation. Lastly, video kinematic analysis unveiled no discernible changes in cardiac contractility, energy and force (Fig. S2A, S2B and S2C) while an increase in trajectory perimeters of the cardiac cycle was observed after PM-Mi treatment (Fig. S2D).

### EGs machine learning performance

The machine learning step revealed a great capability in distinguishing control and PM-treated EG of pregnant rats (Fig. S3). In the NR group, the F1 score was 0.8421, the accuracy was 0.8636 and the AUC score was 0.9337. On the other hand, in the SHR group, the F1 score was 0.7222, the accuracy was 0.7727, and the AUC score was 0.8391. Moreover, if we considered only the exposure to PM-Mi independently to the hypertension, the F1 score was 0.8043, the accuracy was 0.7955 and the AUC score was 0.9021.

### Gene expression and inflammatory protein quantification in pregnant rats

**Fig. 6:**
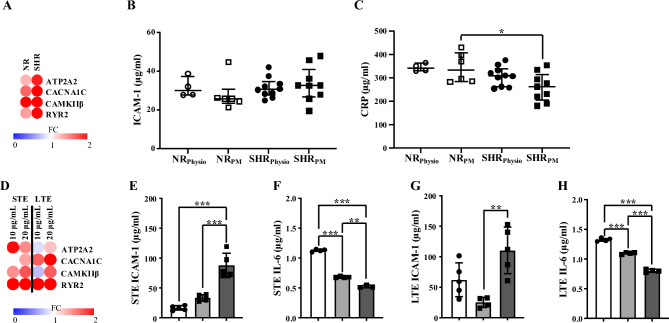
Gene expression and inflammatory markers analysis in Physio (circles) and PM-Mi (squares) treated NR (empty symbols) and SHR (filled symbols) and analysis in cardiac spheroids without PM (control, black circles and white columns) and spheroids stimulated (black squares) with 10 μg/ml of PM-Mi (light gray columns) and 20 μg/ml of PM-Mi (dark grey columns). **A** Comparison of gene remodeling occurring at the level of the listed genes. Left: concerning pregnant normotensive rats treated with PM-Mi (NR), right: concerning pregnant hypertensive rats treated with PM-Mi (SHR). FC: fold change compared to physio treatment, **B** Expression of ICAM-1, **C** Expression of CRP, **D** Comparison of gene remodeling occurring at the level of the listed genes. Left: concerning short term evaluation (STE), right: concerning long-term evaluation (LTE). FC: fold change, **E** Expression of ICAM-1 at short term period, **F** Expression of IL-6 at short term period, **G** Expression of ICAM-1 during long-term period. H) Expression of IL-6 during long-term period. Data were presented as median ± interquartile range and were analyzed by Kruskal–Wallis test. * *p* < 0.05, ** *p* < 0.01 and *** *p* < 0.001

The qPCR analysis revealed marked upregulation of all genes involved in transient calcium pathways (Fig. 6A, Table S2). Comparing the results obtained, there is a marked upregulation of the genes: *Atp2A2*, *Cacna1C*, *CamkIIβ* and *Ryr2* in PM-treated SHR pregnant rats. In PM-treated NR rats, however, there is a milder modulation of the first four genes, while a non-modulation results in the expression of *Gata4* (data not shown). The t-test was significant in all samples considered. assay did not reveal differences in ICAM-1 concentration (Fig. 6B). CRP concentration resulted reduced in SHR_PM_ compared to NR_PM_ (Fig. 6C). IL-6 was not detectible and thus the data are not shown.

### Spheroid functional analysis

**Fig. 7:**
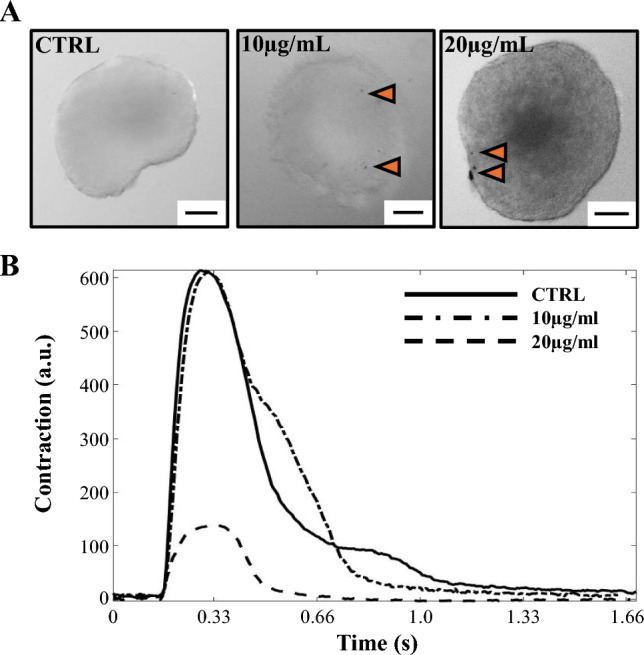
Light microscope images and contractility traces of spheroids after 192 h of PM-Mi exposure. **A** Examples of cardiac spheroids w/o treatment (CTRL) or after PM administration at a concentration of 10 μg/ml and 20 μg/ml. Scale bar 100 μm. Orange arrowheads highlighted PM-Mi localization, **B** Representative profiles of contraction obtained at 192 h exposure timepoint. Solid line: control; dashed-points line: 10 μg/ml, dashed line: 20 μg/ml

PM agglomerates are present (Fig. 7A), indicating intimate interaction between the pollutant and the samples. Throughout the 192 h of monitoring, the contractility of the samples was assessed, and representative contraction profiles are reported (Fig. 7B).

**Fig. 8:**
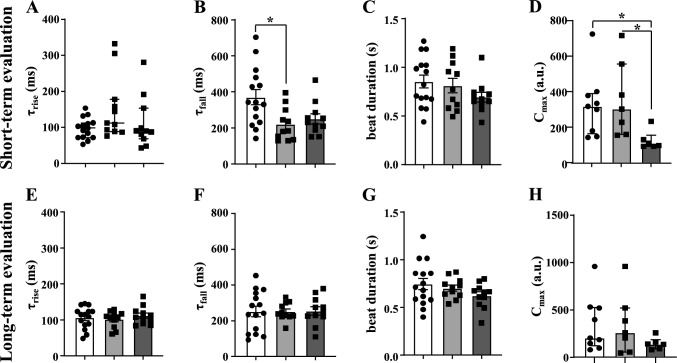
Kinematic analysis of spheroids without PM-Mi (control, black circles and white columns) and spheroids stimulated (black squares) with 10 μg/ml of PM-Mi (light grey columns) and 20 μg/ml of PM-Mi (dark grey columns). Short term evaluation is the response within 48 h of PM-Mi administration. Long-term evaluation is the response between 168 and 192 h of PM-Mi administration. T_rise_ (**A** and **E**) represents the time of contraction from 0 to 90% of the contraction part of the contractility trace. T_fall_ (**B** and **F**) represents the time of relaxation from 100 to 10% of the relaxation part of the contractility trace. Beat duration (**C** and **G**) is the duration of the total beat (contraction and relaxation). C_max_ is the maximum amplitude value of the contraction. Data were presented as median ± interquartile range and were analyzed by Kruskal–Wallis test t. * *p* < 0.05

The profiles of the spheroids showed that both beat duration and amplitude were progressively reduced until a dampened signal is recorded at 50 μg/mL PM-Mi (data not shown), compared to the control. Since the highest concentration of 50 μg/mL resulted in arrhythmic or absent kinematic activity, 10 μg/mL and 20 μg/mL were the PM-Mi concentrations studied thereon. Considering the effect on kinematics in short term evaluation (data pooled from the first 48 h., cf. Fig. S4), the τ_rise_ and beat duration were not altered (Fig. 8A and C). Conversely, the τ_fall_ showed a decreasing tendency (Fig. 8B) turning significant at 10 μg/mL during short term evaluation (control: 343.7 ± 248.2; 10 µg/mL: 183.9 ± 165.6), confirmed by longitudinal analysis at 48 h (Fig. S4B). The amplitude of contraction, C_max_ (control: 317.1 ± 226.3; 20 µg/mL: 103.0 ± 63.5) was significantly reduced in the 20 µg/mL short term evaluation samples (Fig. 8D). In the long-term evaluation (LTE, data pooled from 96 and 192 h), no differences could be appreciated (Fig. 8E, F, G and H, Fig. S4 C–D).

**Fig. 9:**
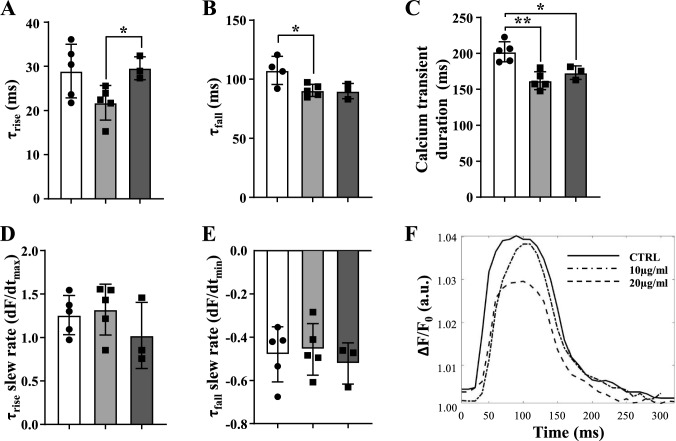
Kinematic analysis of the calcium release and uptake at the end of the PM-Mi treatment (192 h) of spheroids without PM-Mi (control, black circles and white columns) and spheroids stimulated (black squares) with 10 μg/ml of PM-Mi (light gray columns) and 20 μg/ml of PM-Mi (dark grey columns). **A** Duration of calcium release, **B** Duration of calcium reuptake, **C** Total calcium transient duration, **D** Velocity of calcium releasing, **E** Velocity of calcium reuptake, **F** Calcium transient profiles of control (solid line), of spheroids after 10 µg/ml (dashed-points line) and after 20 µg/ml (circles) of PM exposure. Data were presented as mean ± SD range and were analyzed by one-way ANOVA test. * *p* < 0.05 and ** *p* < 0.01.-Mi

Further possible functional differences were inspected by calcium transient analysis (Fig. 9). The calcium release (τ_rise_) in spheroids treated with 10 μg/mL was lower compared to the spheroids treated with 20 μg/mL (10 µg/mL: 21.8 ms ± 3.9; 20 µg/mL: 29.6 ms ± 2.6; Fig. 9A), and reduced the time of calcium reuptake (τ_fall_) in spheroids treated with 10 μg/mL compared to controls (control: 107.4 ms ± 11.9; 10 µg/mL: 90.6 ms ± 5.2; Fig. 9B). Both concentrations modified significantly the calcium transient duration compared to the control (control: 202.2 ms ± 14.1; 10 µg/mL: 162.1 ms ± 12.4; 20 µg/mL: 173.3 ms ± 9.4; Fig. 9C). Lastly, calcium release and reuptake slew rate were not affected (Fig. 9D and E, respectively). Representative fluorescence calcium transient profiles are reported in Fig. 9F.

### Expression of calcium handling-related genes in cardiac spheroids

Similar to the *in-vivo* data, the qPCR analysis (Fig. 6D) revealed a trend of increased transcription of genes involved in calcium transport, particularly when spheroids were treated with 20 µg/mL PM-Mi in both short- and long-term evaluation. Notably, the *Cacna1C* gene showed a gradual increase as a function of both exposure time and PM-Mi concentration. Additionally, the expression of *Ryr2* was significantly increased. The *Atp2A2* gene appeared to be upregulated overall, and the t-test indicated a significant difference in expression in spheroids exposed to both PM-Mi concentrations in the short term, which was also detectable in the LTE at the 20 µg/mL concentration. The trend for *CamkIIβ* gene expression mirrored that of the other genes. In this case, the t-test was significant only in samples exposed to 20 µg/mL in the short-term group. ELISA assay on spheroid supernatant revealed differences both in STE and LTE (Fig. 6E–H). Specifically, ICAM-1 concentration resulted statistically significant higher in 20 µg/mL compared to both control and 10 µg/mL in short term evaluation (Fig. 6E). CRP did not result different in the three experimental groups (Fig. S5A), contrarily to IL-6 down expressed significantly in 20 µg/mL (vs control, Fig. 6F). Long-term exposed samples were observed to preserve higher ICAM-1 concentration in 20 µg/mL compared to the 10 µg/mL (Fig. 6G), whilst CRP was not altered in any PM-Mi exposure conditions (Fig. S5B). Lastly, IL-6 differences were stressed with higher significant differences among all the groups (Fig. 6H). Moreover, IL-6 results altered between time of evaluation, incrementing in all the experimental groups from STE to LTE (Fig. S5C).

## Discussion

The hypothesis that gestational particulate matter (PM) exposure exerts cardiac effects on fetuses of hypertensive mothers is compelling, yet mechanistically complex. Accurately assessing this relationship requires integrated modeling of both maternal pregnancy physiology and dynamic fetal cardiac monitoring. It is well known that an increasing number of respiratory and cardiovascular diseases has been correlated with PM exposure. However, the ability of PM to penetrate tissues depends on several factors, including the geographical region (Yu et al. 2023), season and exposure time, and obviously on its intrinsic physico-chemical properties. The INSIDE project (Ferrari et al. 2020, 2022) addressed this gap by enrolling pregnant women in Milan across seasonal extremes (winter/summer) and quantifying exposure to PM_10_, PM_2.5_, and PM₁. Within this cohort, we identified a significant association between PM_2.5_ exposure and altered fetal heart rate—suggesting autonomic disruption (Iodice et al. 2018). However, elucidating PM-Mi direct impact on the physiology for mothers and fetal cardiac tissue remains infeasible without invasive heart analysis.

To overcome this limitation, our group established, within the INSIDE project, complementary experimental models under tightly controlled conditions: a hypertensive pregnancy model using SHR rats with induced gestational hypertension exposed to PM-Mi, and a cardiac model employing embryonic stem cell-derived 3D cardiac spheroids (RUES) to mimic human fetal heart *in-vitro*.

Critically, the biological impact of PM-Mi depends on its physicochemical properties. PM-Mi predominantly derived by secondary and traffic sources (Perrone et al. 2010) exhibits unique traits and agglomeration into nanoporous structures, sustained negative ζ-potential (− 20 to − 35 mV), and polydispersity indices (PDI) of 0.25–0.64, indicating moderate heterogeneity. These properties align closely with diesel exhaust particles (Rossi et al. 2021), which share high heterogeneity in metal composition (e.g., Fe, Cu, Zn, cf. Table S1) and organic components (e.g., PAHs, VOC), alongside reactive surface chemistry that facilitates oxidative stress. The 1.5-fold lower ζ-potential in dH_2_0 versus DMEM10 underscores a possible role of protein coronas in stabilizing PM suspensions (Monopoli et al. 2020).

Our data demonstrate that PM-Mi increases body weight in normotensive animals, consistent with the epidemiological observations linking PM_2.5_ to excess weight in adolescents (Lopez-Gil et al. 2023). While hypertension alone reduces body weight and induces cardiac hypertrophy in our models, we found PM exposure specifically elevates diastolic blood pressure, mirroring our findings in exposed populations (Dvonch et al. 2009).

These results may reveal distinct metabolic and cardiovascular effects of PM-Mi exposure knowing that the adverse effects of PM may be more noxious in frail categories, as pregnant subjects (Blum et al. 2017; Yuan et al. 2023). Regarding cardiac electrophysiology we observed the strongest reduction in excitability (i.e. rheobase and current threshold) and an increment in the effect refractory period accompanied by an increment of the cardiac cycle movement. These data were obtained from SHR rats exposed to PM-Mi compared to unexposed hypertensive controls, revealing synergistic effects between hypertension and air pollution on cardiac pump function (Du et al. 2016). Furthermore, we observed increased arrhythmogenesis, consistent with our recent findings in animals exposed to diesel exhaust particles (Rossi et al. 2021). Notably, while PM-Mi exposure did not alter longitudinal or transverse cardiac conduction velocities, it significantly reduced both the anisotropy ratio and conduction heterogeneity. The 2 mg/kg dose is standard in preclinical studies (e.g., mice, rats) due to allometric scaling principles, which account for metabolic rate differences between species (Rossi et al. 2021, 2019).

Given that conventional ECG parameters showed no significant differences, we investigated whether artificial intelligence combined with network science could reveal PM-related alterations in ECG patterns. We applied network science analysis to EGs acquired during spontaneous sinus rhythm from both NR and SHR exposed animals, transforming the EGs into visibility graphs (Lacasa et al. 2008). Our k-nearest neighbors (k-NN) classifiers demonstrated high accuracy in identifying PM exposure, with 86.36% accuracy for NR-derived EGs and 77.27% for SHR-derived EGs. These results suggest that the conventional ECGs may intrinsically contain hidden information about PM exposure that is only detectable through advanced analytical methods. Cardiac contraction is not exempt from acute PM exposure. Cardiac cycle parameters, obtained through video kinematic evaluation of epicardial movement, demonstrate this effect, which is accompanied by modulation of calcium-handling machinery via SERCA2a, RYR2, and CACNA1C (L-type calcium channel) genes (Dong et al. 2019). This may lead to a shortening of the calcium transient, as observed in our cardiac spheroids. Upregulation of CACNA1C, RYR2, and ATP2A2 suggests an early compensatory enhancement of calcium cycling, with increased Ca^2+^ influx, sarcoplasmic reticulum (SR) Ca^2+^ release, and Ca^2+^ reuptake aimed at preserving contractility under PM-induced stress (Bers 2002; Mattiazzi and Kranias 2014). In cardiac spheroids, short-term PM_2.5_ evaluation preserved beat kinetics despite impaired Ca^2+^ reuptake, consistent with transcriptional compensation, whereas prolonged exposure (192 h) led to reduced Ca^2+^ transients and contractile dysfunction despite sustained gene upregulation, indicating failure of compensatory mechanisms (Anderson et al. 2011; Hasenfuss and Pieske 2002). CAMKIIβ showed only modest or unchanged expression, suggesting limited involvement of Ca^2+^-dependent signaling in the later stages of functional decline.

Inflammatory responses exhibited differential patterns between maternal hearts and embryonic-derived spheroids. In SHR mothers, circulating CRP levels were reduced and ICAM-1 remained unchanged, suggesting a protective systemic adaptation during pregnancy. Conversely, in cardiac spheroids, IL-6 decreased under both short- and long-term PM evaluation, while ICAM-1 increased significantly, indicating early endothelial and inflammatory activation at the cellular level. Together, these findings reveal a complex interplay between calcium handling and inflammatory signaling in response to PM, highlighting both early compensatory mechanisms and eventual functional vulnerability in the developing heart. Peng et al. demonstrated that an IL-6 deficiency ameliorates PM_2.5_-induced inflammation (Peng et al. 2022). However, other studies report increased IL-6 following PM exposure, suggesting that not all PM components exert identical effects (Hamanaka and Mutlu 2018). Multiple limitations of the literature-proposed models have been overcome in measuring longitudinal contractility in *in-vitro* human cardiac cells. We overcome this limitation by employing a customized high-functional analysis together with the ability to discriminate kinematic differences along the time of exposure, thanks to the LOKI system (Burattini et al. 2024). While the magnitude of contraction is affected by PM-Mi, short-term data show a reduction in relaxation time at 10 µg/mL but not at 20 µg/mL, with no significant changes observed in LTE analysis. Conversely, the effect on calcium transient persists, manifesting as reduced calcium reuptake time and consequently shorter transient duration, consistent with the LTE upregulation of CACNA1C and CAMKIIβ.

## Conclusion

Our integrated approach combining hypertensive rat models with embryonic stem cell-derived cardiac spheroids—mimicking human fetal heart—reveals the dual impact of PM-Mi on cardiovascular physiology. Exposure during pregnancy induce calcium dysregulation via SERCA2a/RYR2 alteration, while paradoxically reducing inflammatory markers. Hypertension synergizes with PM to prolong refractoriness and increase arrhythmogenesis, with ML-detected ECG changes confirming subclinical effects. These findings highlight both fetal cardiac vulnerability to pollution and the value of stem cell-derived models in toxicological studies.

## Limitations

While our study provides novel insights into the cardiovascular effects of gestational PM-Mi exposure, several limitations should be acknowledged. First, the use of embryonic stem cell-derived cardiac spheroids, though a valuable model for fetal cardiac development, may not fully recapitulate the complexity of *in-vivo* heart tissue, including cell–cell interactions and hemodynamic forces. Eight days in *in-vitro* exposure does not fully reflect chronic environmental PM exposure. This exposure window was intentionally chosen to assess early and direct functional effects of PM-Mi on cardiomyocytes derived from embryonic-stage human cardiac spheroids, minimizing confounding long-term adaptive processes. While this model does not recapitulate late-stage cardiac development or lifelong exposure, the observed alterations are consistent with reported cardiovascular effects in early life, and future studies will address longer exposure paradigms. Second, while the SHR rat model mimics hypertensive pregnancy, interspecies differences may limit direct translation to human pathophysiology. Third, although we characterized PM-Mi physicochemical properties, real-world exposure involves variable compositions and seasonal fluctuations that our controlled experiments could not entirely replicate. In the current study, PM_2.5_ dose used was above levels expected to occur in realistic human environmental exposures. However, given the differences in species-specificity between rats and humans (e.g., exposure route, particle distribution, clearance, etc.), we did not have the aim of comparing our *in-vivo* experimental doses with human exposure. Notably, although intratracheal instillation is non-physiological and less desirable than inhalation for risk assessment of airborne dust, instillation studies are more appropriate when the toxic potency of precise dose of specific material or mixture should be evaluated. Finally, while ML-enhanced EG analysis revealed hidden exposure signatures, further validation in larger, diverse cohorts is needed to confirm clinical applicability.

## Supplementary Information

Below is the link to the electronic supplementary material.

Supplementary file1 (DOCX 26 KB)

Supplementary file2 (PDF 37 KB)

Supplementary file3 (PDF 93 KB)

Supplementary file4 (PDF 40 KB)

Supplementary file5 (PDF 22 KB)

Supplementary file6 (PDF 141 KB)

Supplementary file7 (PDF 131 KB)

Supplementary file8 (PDF 13 KB)

## Data Availability

The raw data generated in this study are openly available in the Zenodo repository at 10.5281/zenodo.15855060.
