# Supplementary material for: PM2.5 exposure induces functional alterations in pregnant rats heart and in human stem cell derived cardiac spheroids
Source: Arch Toxicol. 2026 Mar 6;100(7):3143–57. doi: 10.1007/s00204-026-04337-8 (PMC13309412; doi:10.1007/s00204-026-04337-8)
Supplement: Supplementary file 1 — Supplementary file1 (DOCX 26 KB) [file 204_2026_4337_MOESM1_ESM.docx]

**PM_2.5_ exposure induces functional alterations in pregnant rats heart and in human stem cell derived cardiac spheroids**

Flavia Bonalumi^1§^, Margherita Burattini^1,2§^, Rosario Statello^1^, Mirko Hu^1^, Minh Long Hoang^1,3^, Nicola Delmonte^3^, Alessia Caputo^1,4^, Barbara Montanini^4,5^, Francesco Paolo Lo Muzio^1,6^, Silvana Pinelli^1^, Paola Mozzoni^1,7^, Jessica Modica^8^, Andrea Cattaneo^9^, Francesca Rossi^10^, Enrico Bergamaschi^11^, Valentina Bollati^12^, Stefano Rossi^1^* and Michele Miragoli*^1,8^

1. Department of Medicine and Surgery, University of Parma, Parma (IT)
2. Department of Surgery, Dentistry, Pediatrics and Gynaecology, University of Verona, Verona (IT)
3. Department of Engineering and Architecture, University of Parma, Parma (IT)
4. Department of Chemistry, Life Sciences and Environmental Sustainability, University of Parma, Parma (IT)
5. Interdepartmental Research Centre Biopharmanet-Tec, University of Parma, Parma (IT)
6. Charité Universitätsmedizin Berlin, Berlin (DE)
7. CERT, Center of Excellence for Toxicological Research, University of Parma, Parma (IT)
8. IRCCS- Humanitas Research Hospital, Rozzano, Milan (IT)
9. Department of Science and High Technology, Università of Insubria, Como (IT)
10. IMEM-CNR Institute, Parma (IT)
11. Department of Sciences of Public Health and Pediatrics, University of Turin, Turin (IT)
12. Department of Clinical Sciences and Community Health, University of Milan, Milan (IT)

^§^ Co-authors

^*^Corresponding authors

Michele Miragoli

Department of Medicine and Surgery, University of Parma

[michele.miragoli@unipr.it](mailto:michele.miragoli@unipr.it)

Co-corresponding

Stefano Rossi

Department of Medicine and Surgery, University of Parma

stefano.rossi@unipr.it

**Supplementary material**

**Methods**

*Cell culturing*

Briefly, cells were washed with Dulbecco’s Phosphate Buffer Solution (DPBS, Gibco™, catalogue # 14190144) and incubated for 5 minutes at 37°C with appropriate volume of 0.5 µM Ethylenediaminetetraacetic acid (EDTA, Invitrogen, catalogue # AM9260G). After removing EDTA, fresh E8 was added to detach the cells from adhesion surface by gentle pipetting. After centrifuging the cell suspension for 5 minutes at 1100 rpm, the pellet was resuspended in 1 mL of fresh E8, and the cell number was determined with a Burcher’s chamber. Cells were plated on Matrigel-coated wells with a concentration of 42000 cell/cm2 in E8 supplemented with ROCK inhibitor (Y-27632, 1:1000 dilution, Selleckchem, catalogue # S1049). After 24hr, the medium was changed to E8 without Y-27632 and refreshed every 2 to 3 days.

*Oestrus phase evaluation*

At the eleventh week of age, the staging of the oestrous cycle was evaluated in Wistar and Spontaneous Hypertensive female rats. Samples collections were carried out with vaginal lavage and specimens were collected at 9 a.m. to reduce variability. To collect cells from the vagina, approximately 0.2 ml of normal saline solution (isotonic) was drawn into a micropipette. The dropper tip was gently inserted into the vaginal orifice to a depth of approximately 5-10 mm, then the saline solution is poured and withdrawn 2 or 3 times. Attention must be taken not to insert the tip too deeply to avoid cervical stimulation. The dropper tips were used only once to prevent contamination between animals. After washing, a small drop of the sample was placed on the slide (smear) and allowed to air dry. To perform Haematoxylin-staining (HE) in wet fixation, the smear was firstly secured with a fixative spray and successively rehydrated. The samples were incubated with HE staining for 60 sec and washed with 4-5 changes of water or until blue dye stopped coming off the slides. Sequentially, counter staining with Eosin was incubated for 90 sec and washed with 4-5 changes of water or until pink dye stopped coming off the slides. The slides were quickly dehydrated through 3 sequential immersions in 5%, 50% and 95% Ethanol solution and cleared in Xylene for 30 sec. Finally, coverslips were mounted with rapid mounting medium (Sigma-Aldrich, Entellan™, catalogue #1079610100). Usually, cells were identified using an x10 objective; however, in some cases the use of higher objectives were necessary (e.g. to better visualize neutrophils). Care must be taken to examine the entire smear as cell types (leucocytes, nucleated epithelial cells and cornified epithelial cells) and numbers may vary in different areas on the slide. When the Oestrus phase was identified as detecting the highest cellular presence represented by leucocytes, the female was immediately mated for 1 day [22].

*Blood pressure measurements*

After 5 days after mating, systolic blood pressure (SBP) and diastolic blood pressure (DBP) were taken by means of Blood Pressure System (Panlab) that provides an easy and reliable technique to measure systemic blood pressure and cardiovascular parameters in rodents without any invasive catheterization. Before the start of the experiment, the animals were trained to enter the restraint apparatus to become accustomed to it and avoid any pressure increase due to the stress of the procedure. The animals rapidly adapted to the procedure and remained calm throughout the measurements. The same researcher, who was kept blind about the purpose of the study, performed all the measurements. The mean of six measurements was used for statistical evaluation.

*Gene Expression Analysis*

qPCR reactions were performed using the QuantiTect SYBR Green PCR Kit (QIAGEN, Hulsterweg, Netherlands) in a final volume of 50 μL, containing 7 ng of cDNA, 0.3 μM of each primer (see Table S2 for oligonucleotide sequences), and 25 μl of 2x QuantiTect SYBR Green PCR Master Mix. Amplification reactions were conducted under the following PCR conditions: 2 minutes at 50°C to allow UNG to eliminate any PCR product containing dUMP from transport contamination, 15 minutes at 95°C for HotStarTaq DNA Polymerase activation, 40 cycles of 15 seconds denaturation at 95°C, 30 seconds annealing at 58°C, and 30 seconds at 72°C for extension. We added an optional 15-second step at 78.5 °C to eliminate any dimer primers formed during the amplification reaction. The hearts of the pregnant rats were ground with a mortar and pestle in liquid nitrogen, and RNA was extracted using RNeasy® Mini Kit (QIAGEN, Hulsterweg, Netherlands). The High-Capacity RNA-to-cDNA™ Kit (ThermoFisher Scientific, Waltham, Massachusetts, United States) was used for the synthesis of cDNA, starting from 365 ng of RNA, following the manufacturers' instructions. qPCR reactions were performed using the PowerUp™ SYBR™ Green Master Mix (ThermoFisher Scientific, Waltham, Massachusetts, United States) in a final volume of 10 μL, containing 10 ng of cDNA, 0.3 μM of each primer (Table S2), and 5 μL of 2X PowerUp™ SYBR™ Green Master Mix. Amplification reactions were conducted under the following PCR conditions: 2 minutes at 50°C for UDG activation, 2 minutes at 95°C for Dual-Lock™ DNA polymerase activation, 40 cycles of 15 seconds denaturation at 95°C and 1 minute annealing/extension at 60°C.

*Network science and machine learning classification*

Network Science aims to find complex relationships and structures within data by applying principles of graph theory. In particular, the visibility graph algorithm transforms a time series into a graph. Therefore, if we consider an electrograms (EG) as a succession of bars spaced by regular intervals and as high as the signal amplitude, a visibility graph can be obtained by connecting each bar to all the bars that can be reached through a straight line and without intersecting any additional bars (see Equation 1).

$$y_{k}<y_{j}+\left( y_{i}-y_{j} \right)\frac{t_{j}-t_{k}}{t_{j}-t_{i}}$$

Equation 1.

Where $y$is the amplitude of the signal, and $t$is the time point.

After obtaining the graphs from the EG signals, we partitioned them into groups of nodes based on the connectivity of each graph by applying the Community detection algorithm. Following this algorithm that is based on the maximization of the modularity $Q$ (see Equation 2), nodes were tentatively inserted or removed from a community depending on their connections (maximizing the connections within the modules and minimizing those outside the module).

$$Q=\sum_{s=1}^{m} \left( \frac{l_{s}}{L}-\left( \frac{d_{s}}{2L} \right)^{2} \right)$$

Equation 2.

Where $m$is the number of modules, $l_{s}$ is the number of edges within module $s$, $L$is the total number of edges in the network, and $d_{s}$ is the sum of the degrees of the nodes in module $s$.

Depending on the within-module degree $z_{i}$ (see Equation 3), which describes how a node is connected to the other nodes of a graph, and the participation coefficient $P_{i}$ (see Equation 4), which describes the position of a node inside the community, the nodes were classified into roles.

$z_{i}=\frac{\kappa_{i}-\overline{\kappa}_{s_{i}}}{\sigma_{\kappa_{s_{i}}}}$

Equation 3.

$$P_{i}=1-\sum_{s=1}^{m} \left( \frac{\kappa_{is}}{k_{i}} \right)^{2}$$

Equation 4.

Where $\kappa_{i}$ is the number of edges node $i$ has within its module $s_{i}$ , $\overline{\kappa}_{s_{i}}$ is the average internal degree of all nodes in module , $\sigma_{\kappa_{s_{i}}}$ is the standard deviation, $k_{i}$ is the total degree of node $i$.

The percentages of each role were used as features for the machine learning model based on a k-nearest neighbors (k-NN) classifiers.

This classification approach has been separately performed on: i) sinus rhythm EGs recorded from all animals regardless of hypertension (i.e., strain differences); ii) sinus rhythm EGs recorded from normotensive rats; iii) sinus rhythm EGs recorded from spontaneously hypertensive rats.

Three different metrics/indexes were adopted to evaluate the classification performances. Accuracy and F1-score were calculated for a 2x2 confusion matrix (Table 1) as follows:

Accuracy:

$$Accuracy= \frac{TP+TN}{TP+TN+FP+FN}$$

F1-score:

$$F1=2*\frac{precision*recall}{precision+recall}$$

where

$$precision= \frac{TP}{TP+FP}$$

and

$$recall=\frac{TP}{TP+FN}$$

where TP is true positive, TN is true negative, FP is false positive and FN is false negative.

The Area Under the Receiver Operating Characteristic (ROC) curve (AUC) was used to summarize the ability of our binary classifier to discriminate between classes across all possible decision thresholds. Specifically, AUC ranges from 0 to 1, with larger values indicating better classifier performance.

**Supplementary Figure Legends**

**Table S1.** Total Chemical composition of PM-Mi

**Table S2.** Oligonucleotide sequences

**Figure S1.** Blood pressure evaluation. A: Systolic blood pressure (SBP) in Physio (circles) and PM-Mi (squares) treated animals. B: Diastolic blood pressure (DBP) in Physio (circles) and PM-Mi (squares) treated animals. C: blood pressures (BP) in normotensive rats (NR). D: blood pressures (BP) in spontaneously hypertensive rats (SHR). Data were presented as median ± SD range and were analyzed by two-way ANOVA. * p<0.05 and *** p<0.001.

**Figure S2.** Kinematic parameters in Physio (circles) and PM-Mi (squares) treated animals. A) Maximum contraction velocity (Contractility). B) Energy. C) Cardiac fatigue (Force). D: Perimeter normalized on number of beats. NR: normotensive rats. SHR: spontaneously hypertensive rats. Data were presented as median ± interquartile range and were analyzed by two-way ANOVA. * p<0.05.

**Figure S3. Machine Learning performances on EG of pregnant rats**. **A)** Confusion Matrix of NR prediction among two classes: physio and PM-Mi. B) Confusion Matrix of SHR prediction among two classes: physio and PM-Mi. C) Confusion Matrix of NR and SHR prediction among two classes: physio and PM-Mi,. D) NR and SHR ROC curve of classification. ROC: Receiver operating chararcteristic: AUC: Area under curve.

**Figure S4.** Kinematic analysis of spheroids at 24, 48, 168 and 192 hrs. respectively in the presence of 10 and 20 μg/ml of PM-Mi. A: Time of contraction (τ_rise_); B: Time of relaxation (τ_fall_); C: Beat duration; D: maximal contraction amplitude. Data were presented as media ± SD and were analyzed by two-way ANOVA test. * p<0.05.

**Figure S5.** ELISA assay on cardiac spheroids supernatant without PM-Mi (control, black circles and white columns) and spheroids stimulated (black squares) with 10 μg/ml of PM-Mi (light gray colunms) and 20 μg/ml of PM-Mi (dark grey columns). A) CRP in the short-term evaluation (STE) and B) long-term evaluation (LTE). C) Expression of IL-6 at STE and LTE. Data were presented as mean ± SD range and were analyzed by two-way ANOVA. * p<0.05 and **** p<0.0001.
