# Supplementary material for: PM2.5 exposure induces functional alterations in pregnant rats heart and in human stem cell derived cardiac spheroids
Source: Arch Toxicol. 2026 Mar 6;100(7):3143–57. doi: 10.1007/s00204-026-04337-8 (PMC13309412; doi:10.1007/s00204-026-04337-8)
Supplement: Supplementary file 2 — Supplementary file2 (PDF 37 KB) [file 204_2026_4337_MOESM2_ESM.pdf]

Anions (%)		Cations (%)		Elements (%)	
NO_3^-	22.6	NH_4^+	13.9	Fe	0.38
SO_4^{2-}	5.68	Ca^{2+}	2.58	Zn	0.24
Cl^-	1.56	Na^+	2.25	Cu	0.028
		K^+	1.14	Mn	0.014
		Mg^{2+}	0.46	Sr	0.007
				Pb	0.006
				Ni	0.003
				Cr	0.003
				V	0.003
				Se	0.002
				As	0.001
				Cd	0.0003
				Co	0.0003
				Ag	0.0002
