# Supplementary material for: PM2.5 exposure induces functional alterations in pregnant rats heart and in human stem cell derived cardiac spheroids
Source: Arch Toxicol. 2026 Mar 6;100(7):3143–57. doi: 10.1007/s00204-026-04337-8 (PMC13309412; doi:10.1007/s00204-026-04337-8)
Supplement: Supplementary file 3 — Supplementary file3 (PDF 93 KB) [file 204_2026_4337_MOESM3_ESM.pdf]

**Gene**	**Forward**	**Reverse**
Rat *Atp2a2*	GAAACTACCTGGAGCCTGCAAT	AGGGCTGGAAGATGTGTTGC
Rat *Cacn1c*	ATGACTGCTTACGGGGCTTT	TGATCGCGCTGGACTGAATG
Rat *CamkIIβ*	GGAGTCAAGCCCCAGACAAA	CTGTCGGAAGATTCCAGGGC
Rat *Ryr2*	GTCTGGGTGGGCTGGATTAC	CTGCGTTTGATGCTCTCGTG
Human *ATP2A2*	CGTCGGGGAAGTTGTCTGTATT	ACCGTCAGCAGCAATGAACC
Human *CACNA1C*	CTTTGCCTTCGCCATGCTCA	TAGCCCATAGCGTCCTGCAT
Human *CAMKIIβ*	TGAGGCCTACGCGAAAATCT	GGGTTCAGGATGGTCGTGTG
Human *RYR2*	ATCATGCAGCCAGAGCCGAA	AAATACCACCGTCCGGCCTT
