# Supplementary figures and images for: PM2.5 exposure induces functional alterations in pregnant rats heart and in human stem cell derived cardiac spheroids
Source: Arch Toxicol. 2026 Mar 6;100(7):3143–57. doi: 10.1007/s00204-026-04337-8 (PMC13309412; doi:10.1007/s00204-026-04337-8)

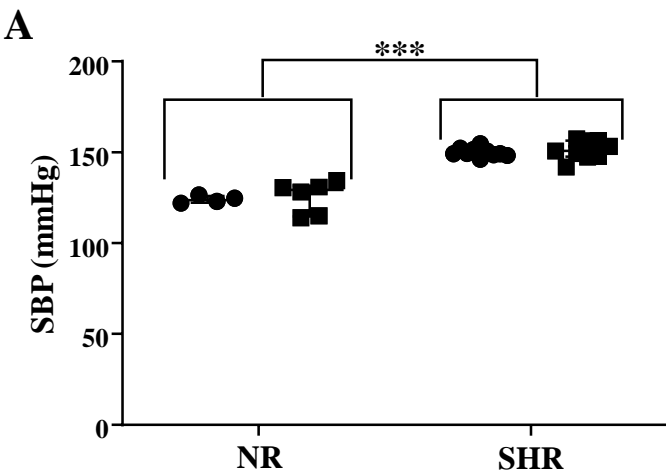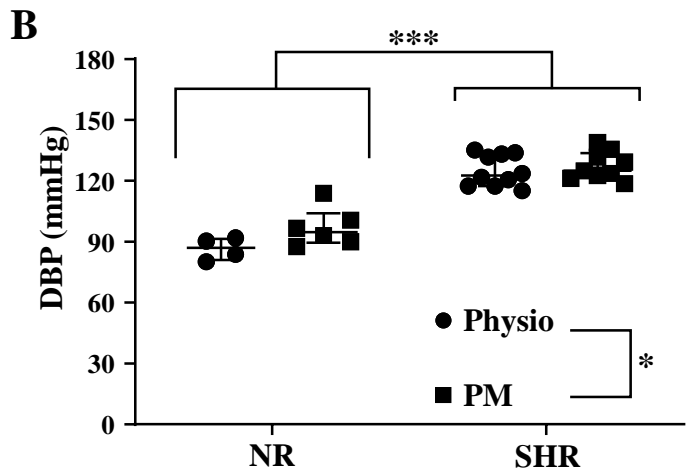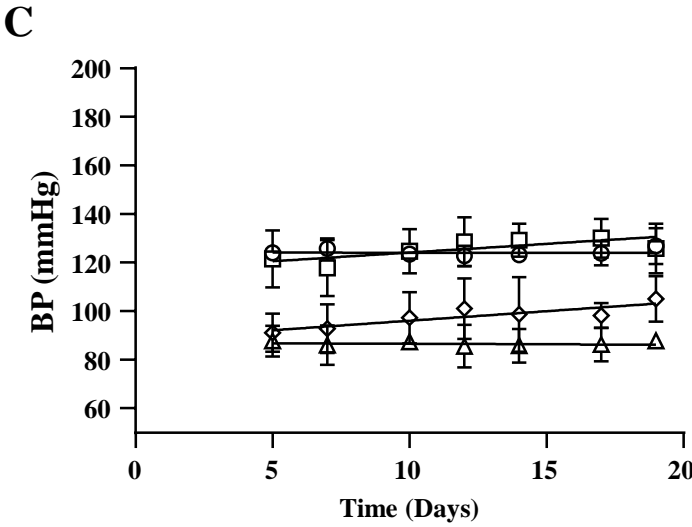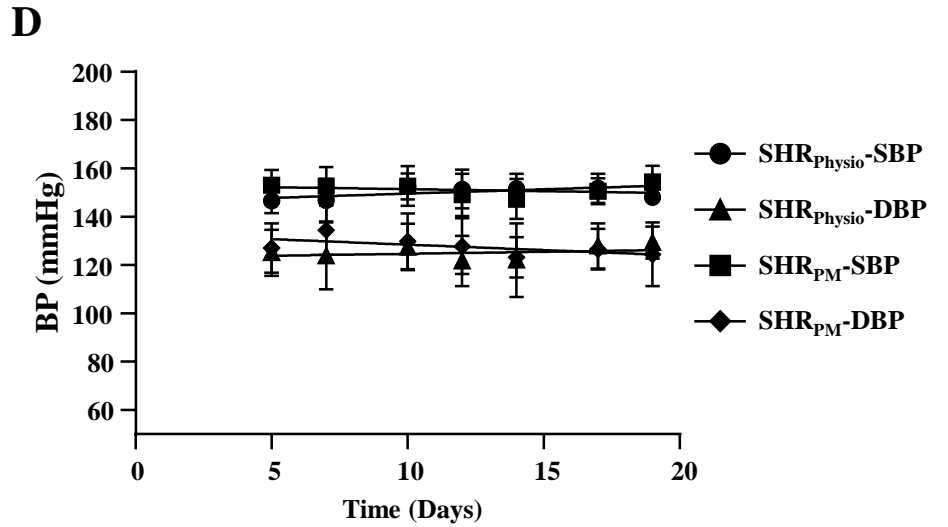

Supplement: Supplementary file 4 — Supplementary file4 (PDF 40 KB) [file 204_2026_4337_MOESM4_ESM.pdf]

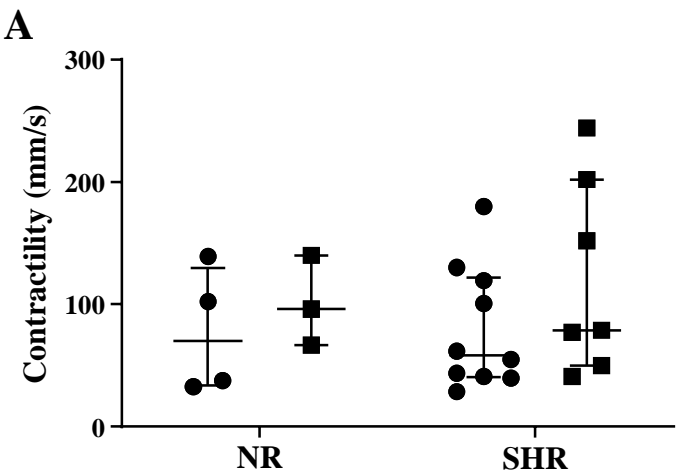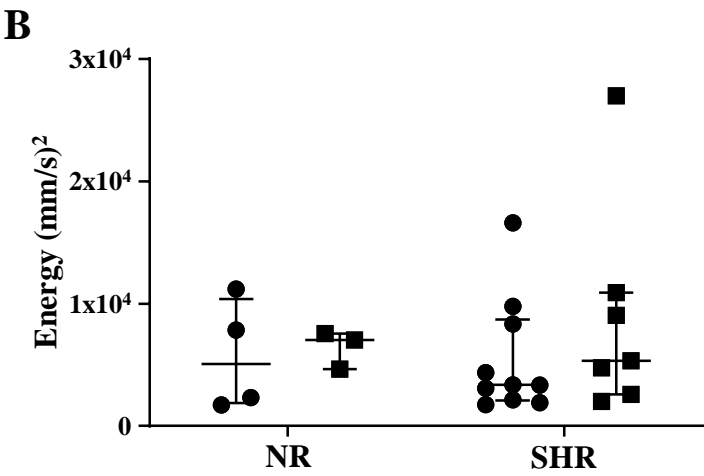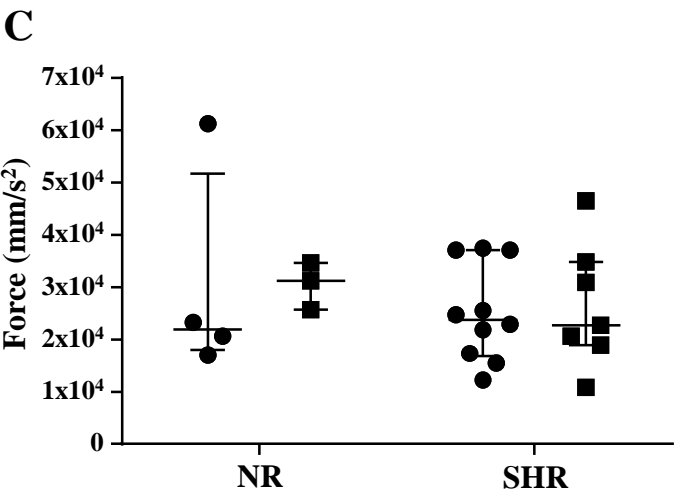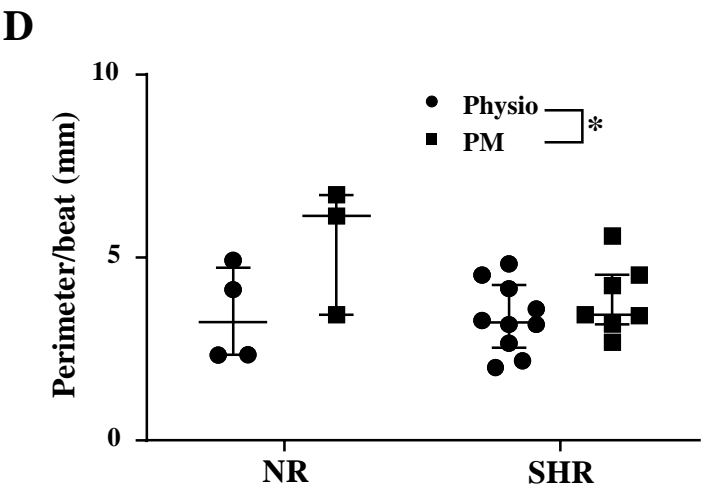

Supplement: Supplementary file 5 — Supplementary file5 (PDF 22 KB) [file 204_2026_4337_MOESM5_ESM.pdf]

**A**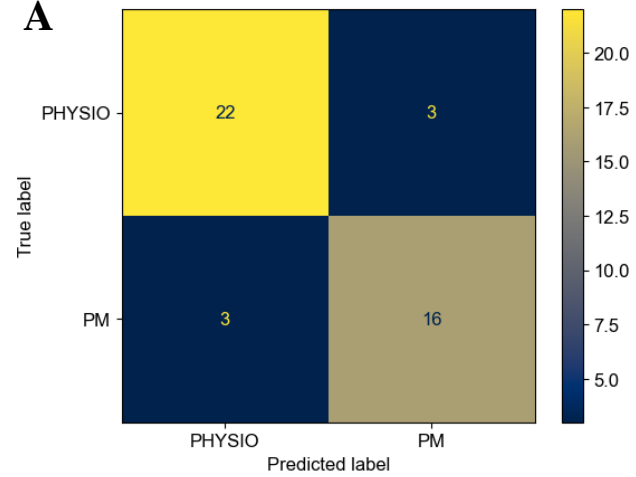**B**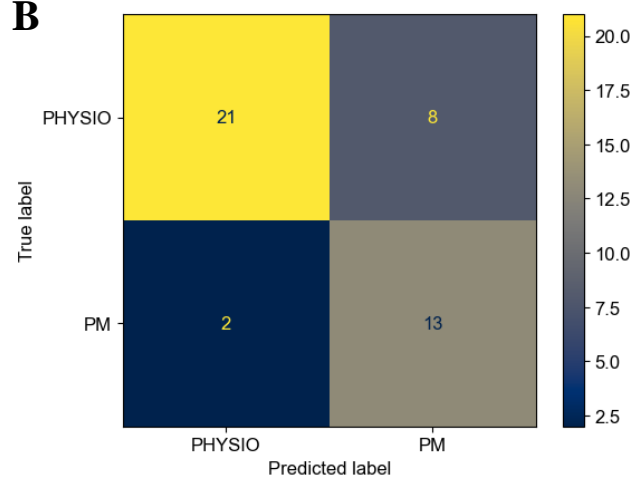**C**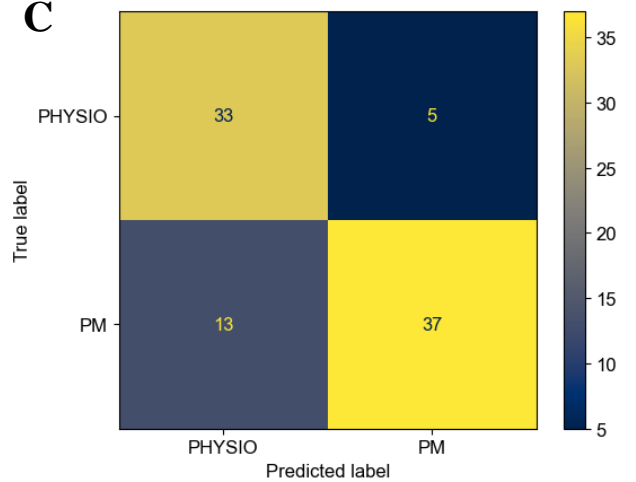**D**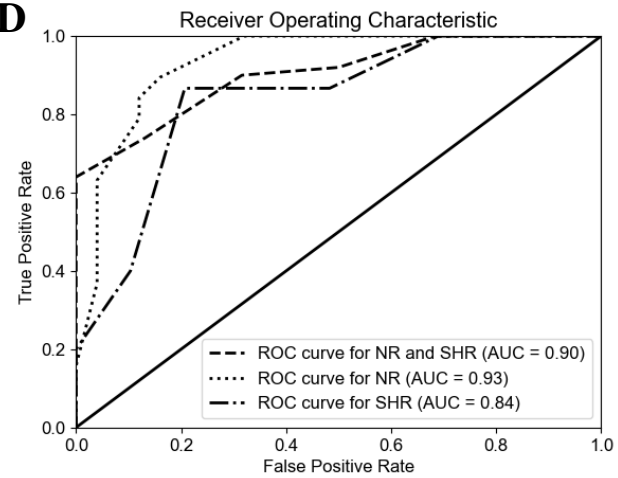

Supplement: Supplementary file 6 — Supplementary file6 (PDF 141 KB) [file 204_2026_4337_MOESM6_ESM.pdf]

**A**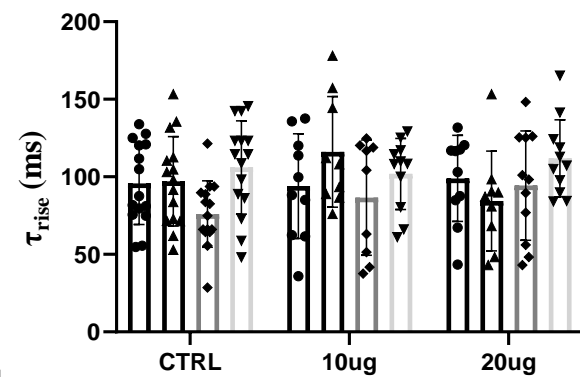**B**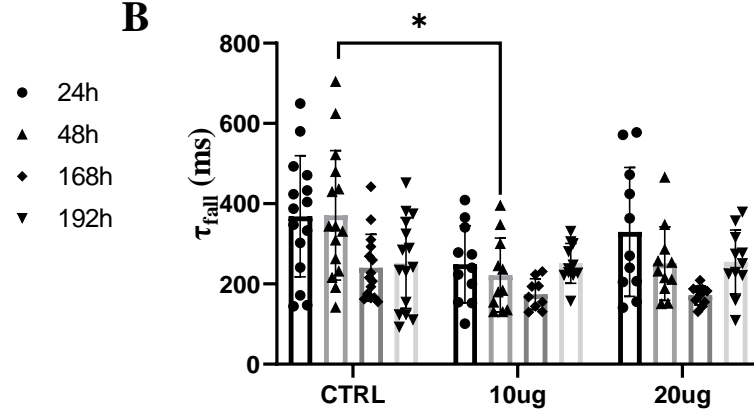**C**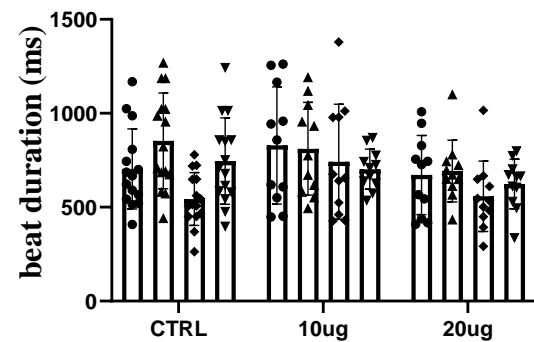**D**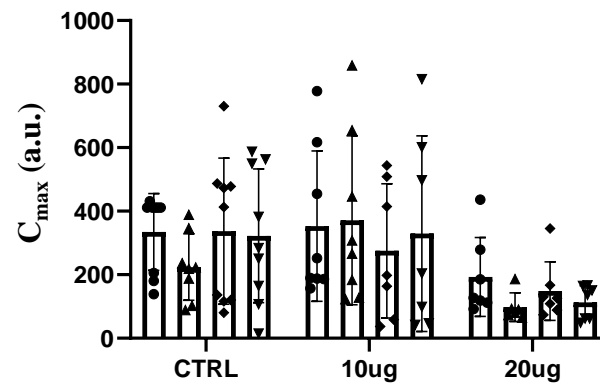

Supplement: Supplementary file 7 — Supplementary file7 (PDF 131 KB) [file 204_2026_4337_MOESM7_ESM.pdf]

Long-term evaluation

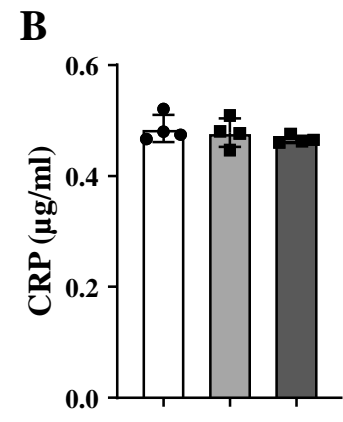

Short-term evaluation

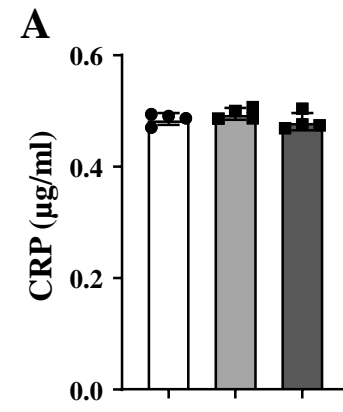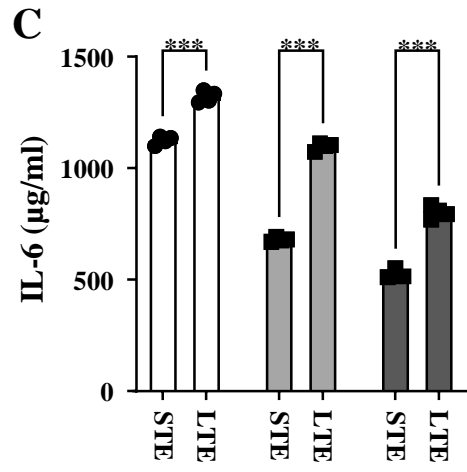

Supplement: Supplementary file 8 — Supplementary file8 (PDF 13 KB) [file 204_2026_4337_MOESM8_ESM.pdf]
